# The Effect of the I-Arch on the Buccal Alveolar Crest in Comparison with the Traditional Archwire Sequence: A Randomized Controlled Clinical Trial

**DOI:** 10.3390/jcm14031026

**Published:** 2025-02-06

**Authors:** Salam Mouhamad Omar Nakawah, Mohamed Hasan Youssef, Ornella Rossi, Giovanna Perrotti, Tiziano Testori

**Affiliations:** 1 Department of Orthodontics, Faculty of Dentistry, Damascus University, Damascus P.O. Box 30621, Syria; salam.nekawe@damascusuniversity.edu.sy (S.M.O.N.); mohamed.youssef@damascusuniversity.edu.sy (M.H.Y.); 2 Department of Biomedical Surgical and Dental Sciences, University of Milan, 20122 Milan, Italy; tiziano.testori@unimi.it; 3 Independent Researcher, Como, Italy; giovanna.perrotti@lakecomoinstitute.com; 4 Department of Implantology and Oral Rehabilitation, Dental Clinic, IRCCS Ospedale, Galeazzi-Sant’Ambrogio, 20157 Milan, Italy; 5 Department of Periodontics and Oral Medicine, School of Dentistry, University of Michigan, Ann Arbor, MI 48109, USA

**Keywords:** alveolar bone height, CBCT, dehiscence and fenestration, dental crowding, I-arch, leveling and alignment

## Abstract

**Background/Objectives**: The nature, diameter, and cross-section of orthodontic archwires affect tooth movement and the surrounding alveolar bone. Researchers have explored different features of archwires to optimize treatment outcomes. In this context, this study aimed to evaluate the properties of the I-arch for its effects on alveolar bone height, dehiscence, fenestration, and treatment duration. **Methods**: Forty patients (eight males, and thirty-two females; mean age: 20.97 ± 2.41 years) with dental crowding ≤ 6 mm and Class I malocclusion were treated without extractions. They were randomly divided into two groups: the experimental group (EG, n = 20), treated with the I-arch, and the control group (CG, n = 20), treated with traditional archwires of the MBT technique. Two CBCT scans were taken for each patient, one before treatment (T0) and one after leveling (T2). The studied teeth were upper and lower centrals, canines, and second premolars. The treatment duration was measured across three periods: T0–T1, T1–T2, and T0–T2. **Results**: Alveolar bone resorption, dehiscence, and fenestration were lower in the EG. Total treatment duration (T0–T2) was similar between groups, but the first period (T0–T1) was significantly shorter in the EG (*p* < 0.05). **Conclusions**: The I-arch resulted in fewer side effects on alveolar bone height during leveling and alignment.

## 1. Introduction

Treatment techniques have been developed in fixed orthodontic devices since the late 18th century by Fauchard [1], Edward Angle, and Andrews [2], and several bracket systems have been proposed, such as MBT brackets, which were developed by Mclaughlin, Bennet, and Trevisi [3,4].

However, fixed orthodontic treatment relates to many side effects like resorption of the alveolar ridge, root resorption, dehiscence and fenestrations, and pain [5,6,7]. Dehiscence and fenestrations are commonly found among orthodontic patients; the dehiscence in the anterior teeth is about 27.46% to 36.51%, whereas the fenestrations are about 26.91% to 51.09% from studied cases [8,9]. The CBCT radiograph, used to investigate bone defects in highly sensitive patients, achieves a detection rate of up to 100% [8] and demonstrates superior accuracy compared to direct clinical diagnosis [10]. One common side effect is alveolar ridge recession, where the height and thickness of the alveolar bone especially in the cervical area decrease during the buccal and lingual movements of the anterior teeth, upper canines, and lower incisors, showing a higher risk of alveolar bone recession in non-extraction cases [11]. A better understanding of biological problems has led to enhanced device designs and treatment methods [12]. One of the most developed materials in orthodontic wires is nickel–titanium [13]. Nitinol is classified into two types: heat-activated NiTi, which does not apply a movement force until a certain temperature is reached by adding small quantities of copper, leading to more constant forces, and providing patient comfort; and super-elastic nitinol, which is low in stiffness causing low-intensity force, so it produces low constant forces [13].

Viazis explains that heat-activated rectangular nitinol archwires can be applied in the first stage of treatment with deficient force magnitude [14,15]. The study by Andreasen showed that the rectangular section of the wire is more able to control the torque of teeth than the square section [16].

Furthermore, the question of treatment duration is one of the first questions asked by patients [17,18], leading to one of the biggest obstacles—patients refusing to undergo treatment [19] because treatment with fixed orthodontic appliances may take 18–30 months [20].

The stages of leveling and alignment are divided into the following two phases: 1—leveling and alignment by round section archwires, accompanied by a buccal crown and lingual root uncontrolled tilting movement [21]; and 2—control roots buccolingual inclination by the rectangular section nitinol archwire, which poses a big challenge for orthodontists, especially in moderate crowding in both dental arches in non-extraction cases [22]. For example, tilting movements often happen and cause resorption of the alveolar ridge caused by protruding teeth during alignment [23]. Other means were explored to provide orthodontists with better control over tooth movement, including modifications to the wire section. One of the recently appeared suggestions is the I-arch, which is considered a creative orthodontic arch wire system, characterized by its vertical cross-section that is longer than the horizontal cross-section [24]. It is claimed that it delivers meager forces during the treatment’s first stage. It is directly applied without applying round wires; therefore, it could decrease the side effects of fixed orthodontic devices by moving crown- and root-deliberated controlled movement, enabling buccolingual inclination control from the beginning of orthodontic treatment, without the need to regain it at later stages. They also claimed that it could save time and reduce the numbers of consumed wires [24]. There is still conflicting information in the literature. Most of the systematic reviews have explained that the scientific evidence concerning the best archwire sequence, its alloy and its cross-section shape, is still limited, and that there is a need for more clinical trials before adopting any cross-section or sequence for the archwires that are studied [21,24,25,26].

Two studies focused on these arch wires: one is an in-vitro study that showed that the NiTi (I-arch) had lower static friction than an old SS wire [27]; however, this study is considered laboratory, so its results cannot be relied upon clinically. The other study is in-vivo, which focused on the effect of the I-arch (0.016 × 0.014 CuNiTi) on anterior teeth by comparing it with a conventional NiTi; it showed that the I-arch was more efficient in alignment in the lower arch and more able to control torque [28]. However, the study has several limitations. The 8-week duration was insufficiently researched, the evaluation of torque expression lacked accuracy, the effects on posterior teeth were not examined, and the results did not distinguish between the upper and lower dental arches, which have different bone structures and may influence the outcomes. Therefore, the information provided remains weak.

The hypothesis of this study is that the use of the I-arch orthodontic archwire, due to its shape and reduced static friction, is more effective than the traditional approach with conventional archwires in reducing side effects during the leveling and alignment phases in cases of dental crowding, and in reducing the overall treatment duration. This study aims to evaluate the efficiency of the I-arch during the leveling and alignment of dental crowding in comparison to the traditional approach followed in conventional archwires in MBT in terms of the investigation of the side effects; these include marginal buccal alveolar bone resorption, and the dehiscence and fenestrations for centrals, canines, and 2nd premolars for both dental arches by studying a CBCT radiograph, in addition to treatment duration during T0–T1, T1–T2, T0–T2.

## 2. Materials and Methods

### 2.1. Study Design

Patients attending the Department of Orthodontics and Dentofacial Orthopedics, Faculty of Dentistry at Damascus University were examined between 1 May 2021 and 30 December 2022. The present study was prospectively recorded at the DRKS—German Clinical Trials Register (ID: DRKS00030098, Registration date: 8 September 2022), with the registration completed prior to the onset of this trial. This two-arm, parallel-group, randomized clinical trial protocol received approval from the Local Research Ethics Committee of the University of Damascus (UDDS-540-24082021GD/SRC-2639). A portion of the study, including the selection of the methodological approach and the statistical analysis, was conducted in collaboration with the Research Center led by Prof. Tiziano Testori in Como, Italy, ensuring rigorous adherence to international research standards and methodological integrity.

### 2.2. Sample Size Estimation

The sample size was calculated using the G*power 3.1.9.4 software presuming that a reduction of 10 percent in total treatment duration could be evidenced with a power of 80 percent at the 5 percent significance level. The statistical test used was the Student’s *t*-test for independent samples (assuming the data were normally distributed).

According to the aims of this study, the sample size was estimated depending on the recession of the alveolar bone crest. The number of patients was 31 based on the Zeitounlouian study [29], and depending on the duration of the leveling and alignment estimates, the number of patients was 36 based on the Eberting study [30].

The highest sample number was taken. Thus, a sample of 36 patients was required for both groups. To account for possible withdrawal, the final sample size for the study was set at 20 patients per group, yielding a total of 40 patients.

**Figure 1:**
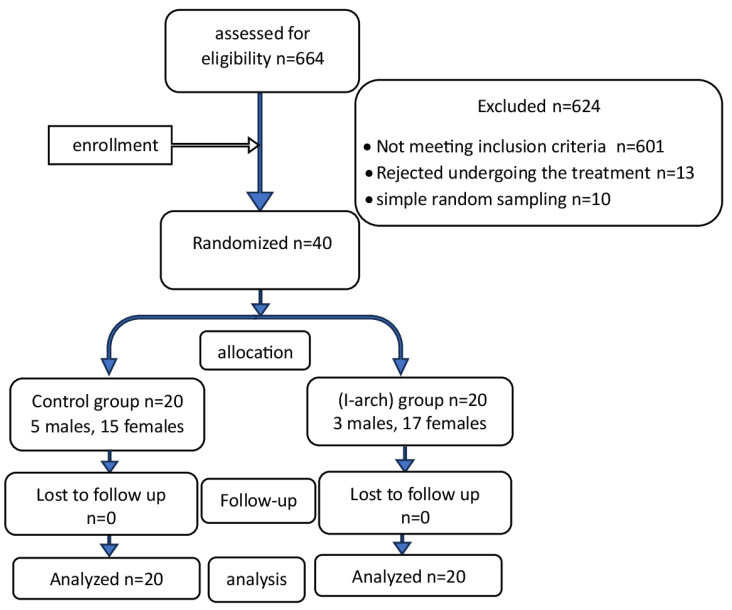
Flow diagram of patients’ recruitment and follow-up.

After the clinical examination of 664 patients at the Department of Orthodontics at the University of Damascus Dental School, it was found that 50 individuals matched the inclusion criteria. In total, 40 of them agreed to take part in the study. Figure 1 explains the flow diagram. Information sheets were provided to all selected patients, then, informed consent forms were obtained.

The inclusion criteria were as follows: (1) adults within an age range of 18–26 years; (2) maxillary and mandibular mild to moderate crowding (≤6 mm) that could be treated without extraction; (3) completion of permanent dentation (except third molars); (4) skeletal class I; (5) molar class I; and (6) the patient has good oral health (according to dental plaque index ≤ 1 mm based on Silness [31]).

The exclusion criteria were as follows: (1) the presence of systemic diseases; (2) medical conditions that would affect tooth movement or gingival tissue or analgesic; (3) sensitivity to orthodontic materials such as nickel–titanium; (4) anterior or lateral crossbite (except presence on one tooth due to crowding); (5) previous orthodontic treatment; (6) skeletal open bite; (7) closed bite obstructs applying braces on lower incisors; and (8) teeth positioned outside the dental arch.

### 2.3. Randomization, Allocation Concealment, and Blinding

Subjects were assigned into two parallel groups with a 1:1 allocation ratio by creating a list of patient names. Every patient had a number, so the list was numbered from one to forty; the odd numbers were in one group and the even numbers were in the other. This procedure was performed by an orthodontist colleague at the Department of Orthodontics not involved in this research. While the blinding of the researcher was not viable, the patients were blinded. The researcher was also blinded during outcomes processing and statistical analysis by covering the patient’s name on the CBCT radiograph.

### 2.4. Treatment Methods

Orthodontic treatment using traditional metal brackets (Master Series^®^, American Orthodontics, Sheboygan, WI, USA) with a 0.018-inch slot high and an MBT prescription was used. Then, the brackets were bonded.

Gr.1 was the I-arch group, and the archwire sequence was 0.016 × 0.014 CuNiTi, 0.018 × 0.014 NiTi [29].

Gr.2 was the control group, and the archwire sequence was as follows: 0.014, 0.016, 0.016 × 0.016, 0.016 × 0.022, 0.017 × 0.025 NiTi [3].

Replacing wires in both groups was accomplished when the used wire became neutral and the next wire could be inserted without applying exaggerated force, indicating complete alignment and easy insertion of the final archwire into all brackets. Interproximal reduction (IPR) was performed to the anterior teeth via diamond stripes for both groups when Bolton’s partial analysis referred to dental–dental disharmony.

### 2.5. Radiographic Study

A CBCT radiograph was taken before the beginning of treatment (T0), and after at least 6 months provided that the leveling and alignment were completed (T2).

The CBCT imaging was performed using the PaX-i3D Green Device (Pax-i3D Green, vatech, Seoul, Republic of Korea), with 5.9 mA, 95 kVp, 15 s exposure time, and an isotropic voxel size of 0.2 × 0.02 mm. All CBCT images were taken in a pose where the Frankfort plane was parallel to the floor [32]. The metal artifact reduction feature was applied in T2 to enhance the radiograph quality. Files were saved in Digital Imaging and Communications in Medicine (DICOM) format; every radiograph had 752 DICOM files, and the images were viewed through “EzDent-i 5.0.2 Simple Viewer Lite” software (Vatech Co., Ltd., Gyeonggi-do, Republic of Korea) [32].

### 2.6. CBCT Radiograph Orientation, Planes, and Related Measurements

**Figure 2:**
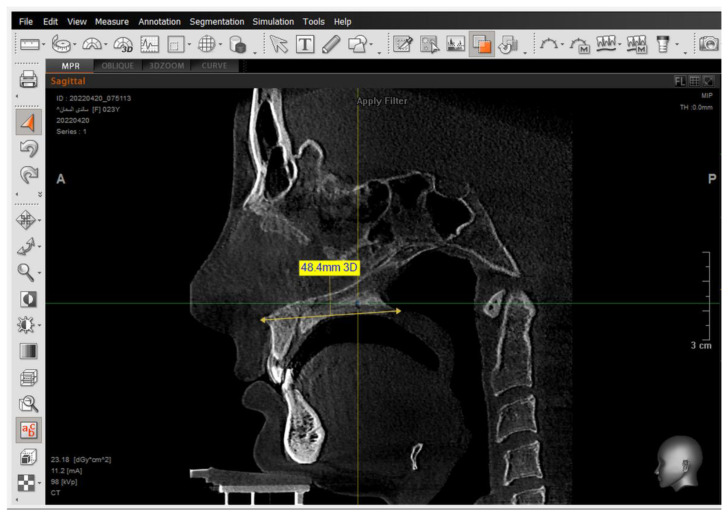
The determination of the maxillary plane (ANS-PNS) in sagittal view, and the selection of the display method to the tissue.

**Figure 3:**
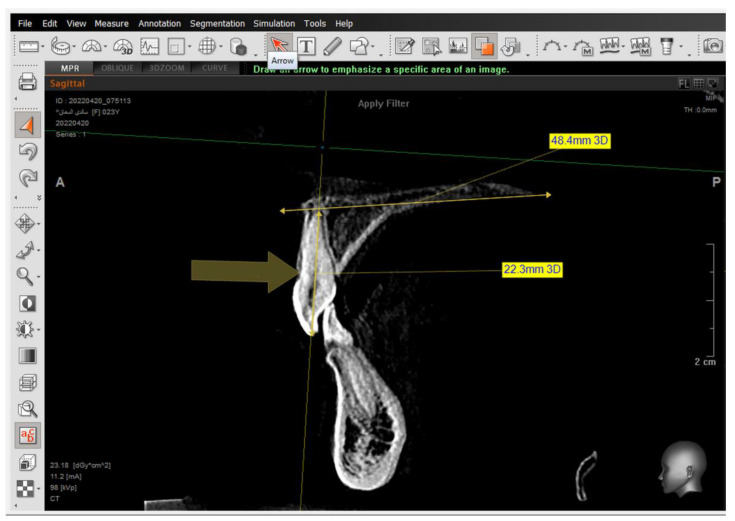
The determination of the long axis of the left upper central 21 in the sagittal view, and the setting of the tip of the buccal alveolar bone crest.

**Figure 4:**
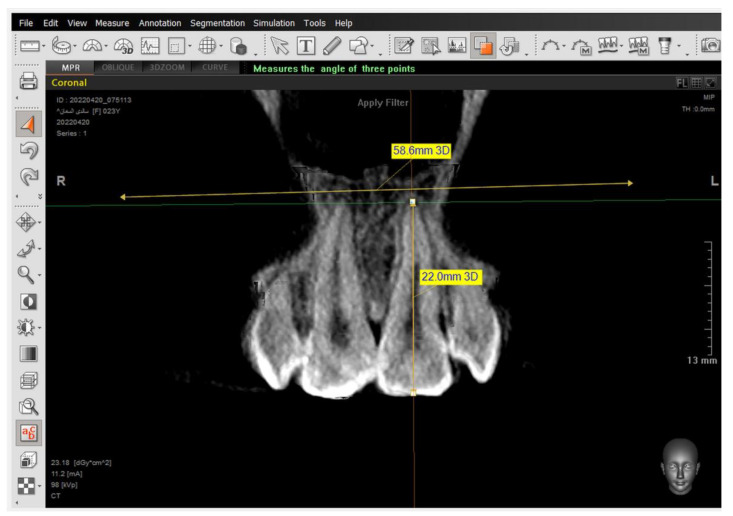
The determination of the long axis of the left upper central 21 in the coronal view.

**Figure 5:**
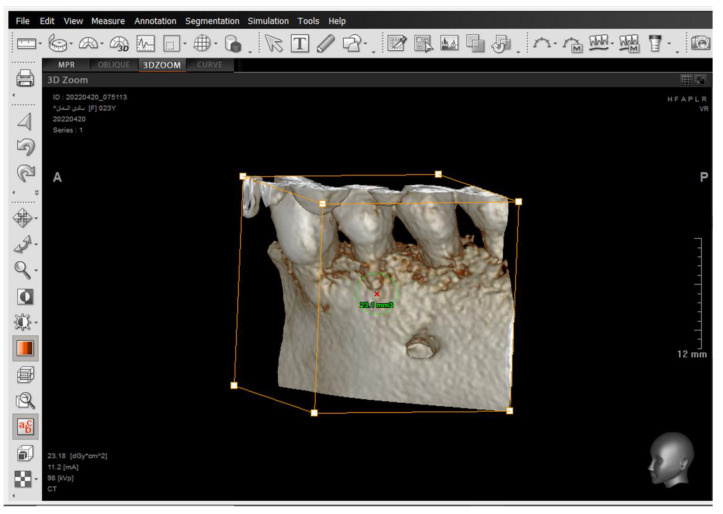
The 3D Zoom tool to accurately investigate the dehiscence and fenestrations.

A 2D-Unsharpen filter was chosen after opening the radiograph to control the clearance of bone borders. The planes that were used and drawn were (ANS-PNS) (Go-Me) in the sagittal view, and (J-J) (Ag-Ag) in the frontal view. The green and yellow (axial and coronal plane) were moved to pass through the axis of the tooth (Figure 2 and Figure 3). Then, the orange sagittal plane in the coronal window was moved until it reached the middle of the incisal margin and the apex of the root (Figure 4). Thus, the largest buccal–lingual section of the centrals and canines was obtained in the sagittal view, referring to the buccal alveolar bone crest, detecting any bony defects (Figure 3). The definition of an alveolar defect when the cortical bone is around the vestibular surface of the root refers to the absence of at least three sequential sagittal views [8]. The defect was confirmed by 3D Zoom when the 2D reading was not clear (Figure 5) [32].

**Figure 6:**
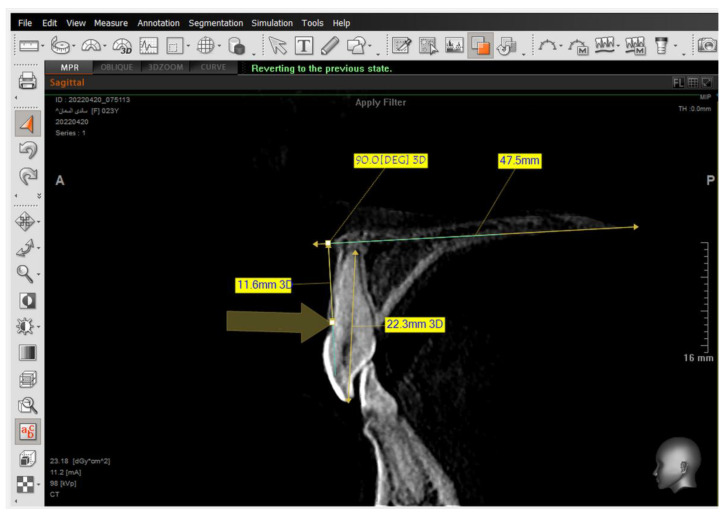
Measuring the distance between the tip of the buccal alveolar bone crest and the maxillary plane, and measuring the angle formed between the tooth longitudinal axis and the maxillary plane in the sagittal view.

Next, a reset to the MPR (Multi-Planar Reconstruction) was performed to measure the distance between the jaw plane and the referred alveolar bone crest (Figure 6) [32].

The same steps were performed for the 2nd premolars but in different directions—in the coronal window for referring buccal alveolar ridge and dehiscence and fenestrations, and in the sagittal window for determining the tooth axis.

### 2.7. Measurements

**Figure 7:**
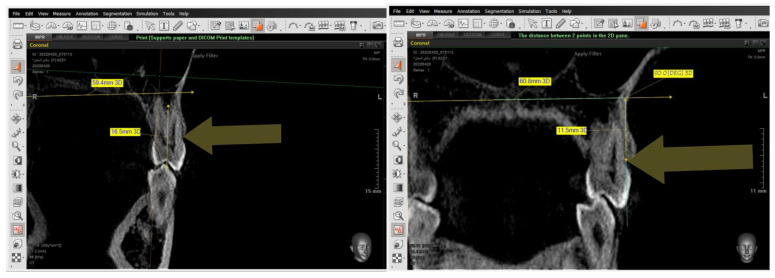
Setting the tip of the alveolar bone crest. Then, measuring the distance between the tip of the buccal alveolar bone crest and the maxillary plane.

The presence of the dehiscence and fenestrations (Figure 5), and the distance between the buccal alveolar crest perpendicular to the reference jaw plane for centrals and canines in the sagittal view were measured (Figure 6), as well as the 2nd premolars in the coronal view in maxilla and mandible (Figure 7) at T0 and T2.

The duration of leveling and alignment was calculated in days during three periods. In the first period (T0–T1), between T0 treatment beginning and T1, the position of the 0.016 × 0.014 CuNiTi archwire was neutral in the I-arch group and the 0.016 × 0.016 NiTi archwire was neutral in the control group. In the second period (T1–T2), between T1 applying (0.018 × 0.014 NiTi in the I-arch group and 0.016 × 0.022 NiTi in the control group) and T2, the archwires were neutral. The third period was the total treatment period (T0–T2) between beginning and finishing the alignment [32].

### 2.8. Statistics Analysis

All measurements were repeated for ten patients after 4 weeks from the first measurement by the same observer. The interclass correlation coefficient ICC was calculated to assess systemic intra-examiner errors between the two measurements. A paired sample *t*-test was used to evaluate validity and to detect any systematic or random error. Dahlberg formula was also applied.

All statistical analyses were performed using SPSS version 25 (IBM Corporation, Armonk, NY, USA), and statistical significance was set at *p* < 0.05.

The Shapiro–Wilk test was used to determine the normality between the measurements at T0 and T2. An analysis of variance was performed to assess changes according to tooth position. To compare two measurements in one group, the paired *t*-test was used for normally distributed samples; for non-normally distributed samples, the Wilcoxon signed rank test was used to reveal any changes in the measures.

To compare between the two groups, for normally distributed samples, the independent *t*-test was used; for non-normally distributed samples, the Mann–Whitney U test was used to reveal any differences in the measures between the two groups.

The Chi-Square Independence Test was used to evaluate the relationship between two qualitative variables.

## 3. Results

### 3.1. The Sample

The sample consists of forty patients: thirty-two females, with a percentage of 80%, and eight males with a percentage of 20%. The age average of the patients in the total sample was 20.97 ± 2.41 years; 20.54 ± 2.42 years in the I-arch group and 21.41 ± 2.41 years in the control group.

### 3.2. The Result of Measurement Accuracy

The second measurements were performed for ten patients at a time interval of one month. The paired *t*-test showed a 95% confidence level for correlated samples and the absence of any significant differences between the two measurements for CBCT variables (Table 1). The method error value according to the Dahlberg formula ranged between 0.09 and 0.28, which means that the systematic and Random errors were minimal. Regarding ICC, the values ranged between 0.91 and 1.00; thus, all measurements had high reliability. Therefore, one measurement reading can be adopted (Table 2).

### 3.3. Study of the Homogeneity of the Two Studied Groups Before Starting Alignment

The results showed the homogeneity of the two studied groups before starting alignment to the dehiscence and fenestrations and the height of the alveolar bone crest. Among the 24 variables, one of them was not homogeneous, which was the buccal alveolar crest of 41 (Table 3 and Table 4).

### 3.4. The Treatment Duration

The total treatment duration (T0–T2) was equal in both groups, whereas the 1st period (T0–T1) was shorter in the I-arch group, and the 2nd period (T1–T2) was shorter in the control group.

### 3.5. CBCT Radiograph Variables Studying for Each Group

#### 3.5.1. I-Arch Group

The decrease in alveolar crest height was significant for three teeth (15, 31, 41), whereas this distance increased significantly in 45 in the I-arch group (Table 5).

The increase in the dehiscence and fenestrations number was significant for three teeth (31, 41, 43) in the I-arch group (Table 6).

#### 3.5.2. Control Group

The decrease in the alveolar bone height happened to all studied teeth, but it was statistically significant in eight teeth (11, 13, 23, 25, 31, 41, 33, 43), while there was no increase in this height in any tooth in the control group (Table 7).

The increase in the dehiscence number and fenestrations was significant for eight teeth (13, 23, 15, 31, 41, 43, 35, 45) in the control group (Table 8).

#### 3.5.3. Comparison Between Two Groups

By comparing the two groups, the height of the buccal alveolar crest decreased in 23, 43, and 45 in the control group more significantly than in the I-arch group (Table 9).

The mean difference of the height decreases in the height of the buccal alveolar crest for all teeth was significantly less in the I-arch group than in the control group (1 mm) (Table 10).

The number of dehiscence and fenestrations increased in 21 and 23 in the control group more than in the I-arch group, whereas it increased in the I-arch group in 43 more than in the control group (Table 11).

The average number of dehiscence and fenestrations of all teeth was significantly less in the I-arch group than in the control group (Table 12).

## 4. Discussion

The buccal alveolar crest recession is considered one of the side effects of orthodontic treatment, which poses a big challenge to the orthodontist, especially to the non-extraction treatment of moderate crowding [22]. These archwires are presented as superior to other wires to avoid side effects, especially in the alveolar crest. However, only one clinical study cared about it.

The treatment duration in the 1st period was less in the I-arch group, and, in contrast, the 2nd period was less in the control group; therefore, the total treatment duration was similar in both groups. It must be noted that the upper teeth alignment is often completed before the lower, but the CBCT radiograph was taken after the alignment of both dental arches was completed. This ensured that the patient would not be overexposed to the radiation, the interval between the two exposures would be at least 6 months, and the CBCT would not be taken unless the last wire was neutral in the two dental arches.

The result of the 1st period was close to Rajan’s study [28], which concerned the 1st archwire 0.016 × 0.014 CuNiTi only. The study found that this I-arch is more efficient in alignment compared to super elastic in the lower arch, and that the values are statistically significant, with the difference in the study duration which was only 8 weeks in the Rajan study. The duration result was consistent with other studies like Atik’s study [33], which did not find a difference in the Little Index between heat-activated nitinol and superelastic nitinol, and Mahmoudzadeh’s study [34], which also compared heat-activated nitinol with super elastic nitinol and did not find a significant difference in the alignment improvement for 4 weeks. What distinguishes this study from the previous two studies is that the 1st archwire in the I-arch group could align teeth alone versus three archwires in the control group. Thus, the 1st archwire was superior in shortening the primary alignment duration by about 44.4 days, eliminating the number of used archwires, and reducing the patient’s time in the clinic without affecting the reduction of dental crowding.

“Bone Traces Tooth Movement” is a basic principle in orthodontics and refers to a high connection between tooth movement and the remodeling of the surrounding alveolar bone to reach the mechanical adaptation in response of the alveolar bone to orthodontic forces [35,36]. The evaluation of the dimensions of the alveolar bone before treatment may help orthodontists in investigating the tooth movement borders and eliminating the risk of iatrogenic occurrence of dehiscence and fenestrations resulting from expansion, retraction, and buccolingual inclination [37]. The remarkable result related to the alveolar crest height in the I-arch group is the increment of the height without any bone grafts in contrast to other studies that showed that the bone augmentation was accompanied by the application of grafts, whether it is autograft, allograft, or synthetic substitutes [38]. Even the decrement of the crest height in three of the twelve teeth in the I-arch group is a phenomenon associated with most fixed orthodontic treatments [39,40]; however, the difficulty lies in reducing the occurrence of this phenomenon. In comparison, the decrement in the control group was eight of twelve teeth. The height decrement is related to the inclination in mild to moderate crowding non-extraction cases, where the greater the increase in the inclination, the greater the buccal alveolar recession [41].

In the comparison of the two groups for all teeth—except molars—the recession of the alveolar bone was less in the I-arch group (1.38 mm) than in the control group. This difference may be explained by the buccolingual inclination control in the I-arch group, and thus may preserve the alveolar bone from reduction.

The method of measuring the alveolar bone recession in this study is similar to El-Mowafi and Nassef’s study [42], which compared rectangular heat-activated archwires versus round heat-activated archwires in leveling and alignment with self-ligating brackets, and where the buccal alveolar bone was measured to the maxillary plane for upper centrals and 1st premolars. The jaw plane was considered as the reference plane in this study because it is constant after growth and more accurate than cementoenamel junction CEJ as a reference point used in other studies. This method is criticized because the tooth could be extruded or intruded during orthodontic treatment so the reference point and the alveolar bone crest is not constant, thus the measurement is performed between two moving points, whereas the measurement method in this study is more accurate because it is performed from a moving point to the constant plane.

The dehiscence and fenestrations are concentrated in anterior teeth in the I-arch group, especially the lower anterior area due to the thinness of the buccal cortical bone, while in the control group, the dehiscence and fenestrations were in the anterior and posterior teeth, and this may be explained by the type of tooth movement resulting from the applied archwire.

This result was similar to the Luo et al. retrospective study [43] of 500 patients; it showed that the leveling and alignment of lower anterior teeth is accompanied by the dehiscence and fenestrations phenomenon.

This study presents several strengths, including the use of CBCT imaging to accurately assess alveolar bone modifications, ensuring reliable and reproducible measurements. The study design also minimized patient exposure to radiation by adhering to strict imaging protocols. Additionally, the comparison of two distinct orthodontic archwire protocols provides valuable insights into their impact on treatment efficiency and alveolar bone preservation, offering clinically relevant information for orthodontists.

However, the study has some limitations that deserve recognition. The sample size was relatively small, which might limit the generalizability of the findings. Furthermore, the follow-up period was restricted to the alignment phase, leaving the long-term effects of the archwire protocols on alveolar bone and dental stability unexplored. The lack of randomization might have introduced selection bias, and the retrospective comparison with other studies could influence the interpretation of the results.

Future studies with larger sample sizes, randomized controlled designs, and extended follow-up periods will help validate these findings and provide a more comprehensive understanding of the effects of different orthodontic archwire systems in orthodontic treatment. Future research should focus on long-term evaluation through longitudinal studies to assess the lasting effects of I-arch wires on alveolar crest preservation and compare them with other orthodontic techniques. Additionally, the efficacy of I-arch wires should be investigated in diverse patient populations, including those with severe crowding and varying skeletal patterns, to enhance their applicability across different cases. Mechanistic studies are needed to explore the biological mechanisms behind alveolar bone remodeling associated with I-arch wires, focusing on their influence on bone cell activity. Innovations in design could involve refining I-arch wire materials and structural design to improve treatment efficiency and bone health. Future studies could also explore the potential of integrating I-arch wires with regenerative techniques to optimize bone health during treatment. Finally, research should examine how I-arch wires affect patient comfort, perceived treatment time, and overall satisfaction to enhance orthodontic care and encourage their adoption in clinical practice.

## 5. Conclusions and Future Directions

The I-arch offers advantages over traditional archwires during the leveling and alignment phase of non-extraction treatment for dental crowding. It is associated with reduced recession of the buccal alveolar crest and fewer bone defects, without compromising treatment duration. Additionally, the I-arch demonstrates superior efficiency in tooth alignment, requiring fewer archwires and reducing overall clinical time, while still effectively addressing dental crowding. These findings suggest that the I-arch is a promising option for improving alveolar bone preservation and optimizing treatment outcomes in non-extraction cases, providing valuable implications for orthodontic practice. Future research should focus on long-term studies to further evaluate the effects of I-arch wires on alveolar bone preservation and treatment outcomes across diverse patient populations and various types of malocclusions.

## Figures and Tables

**Table 1:** The results of the statistical significance tests to study the differences in the mean of the studied measurements on the CBCT images to evaluate systemic errors (N = 20) †.

Variable	Mean for 1st Measure	SD for 1st Measure	Mean for 2nd Measure	SD for 2nd Measure	Differences Between Two Measurements				
					Mean	SD	Standard Error	Probability Value	Sig/NS
Alveolar bone 11	14.225	2.73378	14.12	2.70469	0.105	0.40062	0.08958	0.256	NS
Alveolar bone 21	14.225	2.73378	14.15	2.67945	0.075	0.37258	0.08331	0.379	NS
Deh and fenes 11	0.15	0.366	0.2	0.41	−0.05	0.224	0.05	0.33	NS
Deh and fenes 21	0.1	0.308	0.15	0.366	−0.05	0.394	0.088	0.577	NS
Alveolar bone 13	13.115	2.44976	13.14	2.44053	−0.025	0.12927	0.02891	0.398	NS
Alveolar bone 23	13.15	2.45753	13.195	2.40098	−0.045	0.26453	0.05915	0.456	NS
Deh and fenes 13	0.4	0.503	0.45	0.51	−0.05	0.224	0.05	0.33	NS
Deh and fenes 23	0.35	0.489	0.3	0.47	0.05	0.394	0.088	0.577	NS
Alveolar bone 15	11.315	1.57322	11.27	1.59905	0.045	0.13169	0.02945	0.143	NS
Alveolar bone 25	11.305	1.55444	11.3	1.59638	0.005	0.19324	0.04321	0.909	NS
Deh and fenes 15	0.2	0.41	0.15	0.366	0.05	0.224	0.05	0.33	NS
Deh and fenes 25	0.25	0.444	0.15	0.366	0.1	0.308	0.069	0.163	NS
Alveolar bone 31	29.125	4.09015	29.075	4.13482	0.05	0.13955	0.0312	0.126	NS
Alveolar bone 41	29.185	4.07822	29.085	4.12977	0.1	0.2	0.04472	0.093	NS
Deh and fenes 31	0.5	0.513	0.45	0.51	0.05	0.224	0.05	0.33	NS
Deh and fenes 41	0.45	0.51	0.45	0.51	0	0.324	0.073	1	NS
Alveolar bone 33	27.875	3.99709	27.85	3.96239	0.025	0.32261	0.07214	0.733	NS
Alveolar bone 43	27.875	3.99709	27.855	4.01031	0.02	0.13611	0.03044	0.519	NS
Deh and fenes 33	0.55	0.51	0.55	0.51	0	0.324	0.073	1	NS
Deh and fenes 43	0.5	0.513	0.55	0.51	−0.05	0.394	0.088	0.577	NS
Alveolar bone 35	9.065	4.8489	9.08	4.86509	−0.015	0.09881	0.02209	0.505	NS
Alveolar bone 45	9.065	4.8489	9.14	4.89139	−0.075	0.19433	0.04345	0.101	NS
Deh and fenes 35	0.25	0.444	0.3	0.47	−0.05	0.224	0.05	0.33	NS
Deh and fenes 45	0.2	0.41	0.3	0.47	−0.1	0.308	0.069	0.163	NS

† Paired sample *t*-test is used for paired samples. Deh and fenes: dehiscence and fenestrations. Alveolar bone: distance between buccal alveolar bone crest and jaw plane.

**Table 2:** The results of the statistical significance tests to study the correlation between the two measurements of the studied changes in CBCT images to evaluate random errors via the ICC test, in addition to the Dahlberg test.

Studied Variables	The Number of Repeated Readings	Dahlberg’s Average Error of Measurement	Value F	95 Confidence Level		ICC Intraclass Correlation
				Minimum	Maximum	
Alveolar bone 11	20	0.286401	183.286	0.998	0.986	0.994 ^c^
Alveolar bone 21	20	0.26247	819.481	1.000	0.997	0.999 ^c^
Dehiscence and fenestration 11	20	0.224165	11.105	0.964	0.776	0.910 ^c^
Dehiscence and fenestration 21	20	0.273975	623.573	0.999	0.996	0.998 ^c^
Alveolar bone 13	20	0.090915	1430.140	1.000	0.998	0.999 ^c^
Alveolar bone 23	20	0.185204	3663.441	1.000	0.999	1.000 ^c^
Dehiscence and fenestration 13	20	0.223607	19.526	0.980	0.873	0.949 ^c^
Dehiscence and fenestration 23	20	0.273975	109.414	0.996	0.977	0.991 ^c^
Alveolar bone 15	20	0.09644	579.319	0.999	0.995	0.998 ^c^
Alveolar bone 25	20	0.133231	2788.880	1.000	0.999	1.000 ^c^
Dehiscence and fenestration 15	20	0.158311	11.105	0.964	0.776	0.910 ^c^
Dehiscence and fenestration 25	20	0.224165	3086.498	1.000	0.999	1.000 ^c^
Alveolar bone 31	20	0.183514	3473.027	1.000	0.999	1.000 ^c^
Alveolar bone 41	20	0.155724	1901.902	1.000	0.999	0.999 ^c^
Dehiscence and fenestration 31	20	0.158311	19.947	0.980	0.876	0.950 ^c^
Dehiscence and fenestration 41	20	0.223607	6440.079	1.000	1.000	1.000 ^c^
Alveolar bone 33	20	0.094921	607.716	0.999	0.996	0.998 ^c^
Alveolar bone 43	20	0.094921	1023.639	1.000	0.998	0.999 ^c^
Dehiscence and fenestration 33	20	0.223607	8.900	0.958	0.726	0.993 ^c^
Dehiscence and fenestration 43	20	0.273861	669.468	0.999	0.996	0.999 ^c^
Alveolar bone 35	20	0.12145	619.892	0.999	0.982	0.997 ^c^
Alveolar bone 45	20	0.144049	2511.372	1.000	0.999	1.000 ^c^
Dehiscence and fenestration 35	20	0.158114	15.737	0.975	0.842	0.936 ^c^
Dehiscence and fenestration 45	20	0.223607	7.222	0.942	0.641	0.955 ^c^

^c^: ICC Interclass Correlation test.

**Table 3:** Testing the homogeneity of samples between the two study groups before starting treatment for CBCT radiographic variables according to the studied teeth.

Tooth	I-Arch Group		Control Group		Differences Between the Two Means	*p*-Value
	Mean	SD	Mean	SD		
11 (upper right central)	14.83	2.44	15.12	2.89	−0.29	0.882 ^b^
21 (upper left central)	14.74	2.3	14.95	2.72	−0.21	0.794 ^a^
13 (upper right canine)	14.25	2.86	14.33	3.22	−0.08	0.934 ^a^
23 (upper left canine)	13.71	2.3	13.81	2.22	−0.1	0.561 ^b^
15 (upper right 2nd premolar)	13.22	2.48	12.13	1.68	1.09	0.115 ^a^
25 (upper left 2nd premolar)	13.4	2.76	12.18	1.82	1.22	0.110 ^a^
31 (lower left central)	31.56	3.99	33.32	3.66	−1.76	0.153 ^a^
41 (lower right central)	30.64	4.26	33.32	3.75	−2.68	0.041 ^a,^*
33 (lower left canine)	28.27	3.94	30.09	4.1	−1.82	0.161 ^a^
43 (lower right canine)	28.13	4.19	29.83	3.39	−1.7	0.165 ^a^
35 (lower left 2nd premolar)	9.41	5.31	10.56	4.32	−1.15	0.457 ^a^
45 (lower right 2nd premolar)	9.37	5.31	12.58	9.68	−3.21	0.507 ^b^

^a^: Independent *t*-test, ^b^: Mann–Whitney U test. * significant at the 0.05 level.

**Table 4:** Testing the homogeneity of samples between the two study groups before starting treatment for dehiscence and fenestrations according to the studied teeth.

Tooth	Dehiscence and Fenestrations (T0)	I-Arch Group		Control Group		*p*-Value
		The Number	Percentage	The Number	Percentage	
11 (upper right central)	Presence	20	100.00%	19	95.00%	0.311
	Absence	0	0.00%	1	5.00%	
21 (upper left central)	Presence	17	85.00%	15	75.00%	0.548
	Absence	3	15.00%	5	25.00%	
13 (upper right canine)	Presence	17	85.00%	19	95.00%	0.705
	Absence	3	15.00%	1	5.00%	
23 (upper left canine)	Presence	19	95.00%	18	90.00%	0.49
	Absence	1	5.00%	2	10.00%	
15 (upper right 2nd premolar)	Presence	14	70.00%	9	45.00%	0.292
	Absence	6	30.00%	11	55.00%	
25 (upper left 2nd premolar)	Presence	18	90.00%	19	95.00%	0.548
	Absence	2	10.00%	1	5.00%	
31 (lower left central)	Presence	14	70.00%	17	85.00%	0.256
	Absence	6	30.00%	3	15.00%	
41 (lower right central)	Presence	12	60.00%	14	70.00%	0.376
	Absence	8	40.00%	6	30.00%	
33 (lower left canine)	Presence	16	80.00%	18	90.00%	0.342
	Absence	4	20.00%	2	10.00%	
43 (lower right canine)	Presence	16	80.00%	18	90.00%	0.197
	Absence	4	20.00%	2	10.00%	
35 (lower left 2nd premolar)	Presence	12	60.00%	12	60.00%	0.376
	Absence	8	40.00%	8	40.00%	
45 (lower right 2nd premolar)	Presence	17	85.00%	19	95.00%	0.292
	Absence	3	15.00%	1	5.00%	

T0: before starting treatment.

**Table 5:** The results of the comparison test between the two times before and after leveling and alignment according to the alveolar crest height within the I-arch group according to the studied teeth.

Tooth	T0		T2		Differences Between the Two Means	*p*-Value
	Mean	SD	Mean	SD		
11	14.83	2.44	14.82	2.45	0.01	0.936 ^b^
21	14.74	2.3	14.57	2.23	0.17	0.274 ^a^
13	14.25	2.86	14.18	2.34	0.07	0.782 ^a,^*
23	13.71	2.3	13.73	2.09	−0.02	0.959 ^a^
15	13.22	2.48	12.27	3.35	0.95	0.040 ^a,^*
25	13.4	2.76	12.93	2.42	0.47	0.198 ^b^
31	31.56	3.99	27.92	4.24	3.64	<0.001 ^a,^*
41	30.64	4.26	27.34	4.45	3.3	0.001 ^a,^*
33	28.27	3.94	27.5	4.67	0.77	0.278 ^a^
43	28.13	4.19	27.18	4.5	0.95	0.059 ^a^
35	9.41	5.31	10.65	4.01	−1.24	0.205 ^a^
45	9.37	5.31	11.3	3.9	−1.93	0.012 ^a,^*

^a^: Paired samples *t*-test, ^b^: Wilcoxon signed rank test. * significant at the 0.05 level. T0: before starting the treatment. T2: finishing leveling and alignment.

**Table 6:** The results of the comparison test between the two times before and after leveling and alignment according to dehiscence and fenestrations within the I-arch group according to the studied teeth.

Tooth	Dehiscence and Fenestrations (T0)	Dehiscence and Fenestrations (T2)				*p*-Value
		Absence		Presence		
		The Number	Percentage	The Number	Percentage	
11 (upper right central)	Absence	19	95.00%	1	5.00%	1
	Presence	0	0.00%	0	0.00%	
21 (upper left central)	Absence	16	80.00%	1	5.00%	1
	Presence	1	5.00%	2	10.00%	
13 (upper right canine)	Absence	16	80.00%	1	5.00%	0.07
	Presence	0	0.00%	3	15.00%	
23 (upper left canine)	Absence	19	95.00%	0	0.00%	0.063
	Presence	0	0.00%	1	5.00%	
15 (upper right 2nd premolar)	Absence	10	50.00%	4	20.00%	1
	Presence	4	20.00%	2	10.00%	
25 (upper left 2nd premolar)	Absence	15	75.00%	3	15.00%	0.625
	Presence	1	5.00%	1	5.00%	
31 (lower left central)	Absence	3	15.00%	11	55.00%	0.001 *
	Presence	0	0.00%	6	30.00%	
41 (lower right central)	Absence	2	10.00%	10	50.00%	<0.001 *
	Presence	0	0.00%	8	40.00%	
33 (lower left canine)	Absence	11	55.00%	5	25.00%	0.125
	Presence	0	0.00%	4	20.00%	
43 (lower right canine)	Absence	0	0.00%	16	80.00%	0.008 *
	Presence	0	0.00%	4	20.00%	
35 (lower left 2nd premolar)	Absence	7	35.00%	5	25.00%	0.063
	Presence	2	10.00%	6	30.00%	
45 (lower right 2nd premolar)	Absence	12	60.00%	5	25.00%	0.219
	Presence	1	5.00%	2	10.00%	

* significant at the 0.05 level. T0: before starting the treatment. T2: after finishing leveling and alignment.

**Table 7:** The results of the comparison test between the two times before and after leveling and alignment according to the alveolar crest height within the control group according to the studied teeth.

Tooth	T0		T2		Differences Between the Two Means	T0
	Mean	SD	Mean	SD		
11	15.12	2.89	14.73	2.74	0.39	0.019 ^a,^*
21	14.95	2.72	14.6	2.59	0.35	0.055 ^a^
13	14.33	3.22	13.87	2.97	0.46	0.016 ^a,^*
23	13.81	2.22	12.81	3.51	1	0.010 ^b,^*
15	12.13	1.68	11.6	2.09	0.53	0.134 ^a^
25	12.18	1.82	11.11	2.66	1.07	0.002 ^b,^*
31	33.32	3.66	29.43	4.28	3.89	<0.001 ^a,^*
41	33.32	3.75	29.52	4.21	3.8	<0.001 ^a,^*
33	30.09	4.1	28.12	6.06	1.97	0.042 ^a,^*
43	29.83	3.39	28.35	4.08	1.48	0.006 ^a,^*
35	10.56	4.32	9.44	4.73	1.12	0.177 ^a^
45	12.58	9.68	9.82	4.38	2.76	0.380 ^b^

^a^: Paired samples *t*-test, ^b^: Wilcoxon signed rank test. * significant at the 0.05 level. T0: before starting the treatment. T2: after finishing leveling and alignment.

**Table 8:** The results of the comparison test between the two times before and after leveling and alignment according to dehiscence and fenestrations within the control group according to the studied teeth.

Tooth	Dehiscence and Fenestrations (T0)	Dehiscence and Fenestrations (T2)				*p*-Value
		Absence		Presence		
		The Number	Percentage	The Number	Percentage	
11 (upper right central)	Absence	15	75.00%	4	20.00%	0.375
	Presence	1	5.00%	0	0.00%	
21 (upper left central)	Absence	12	60.00%	3	15.00%	0.07
	Presence	0	0.00%	5	25.00%	
13 (upper right canine)	Absence	12	60.00%	7	35.00%	0.004 *
	Presence	0	0.00%	1	5.00%	
23 (upper left canine)	Absence	11	55.00%	7	35.00%	0.002 *
	Presence	1	5.00%	1	5.00%	
15 (upper right 2nd premolar)	Absence	4	20.00%	5	25.00%	0.016 *
	Presence	1	5.00%	10	50.00%	
25 (upper left 2nd premolar)	Absence	15	75.00%	4	20.00%	0.125
	Presence	0	0.00%	1	5.00%	
31 (lower left central)	Absence	0	0.00%	17	85.00%	<0.001 *
	Presence	1	5.00%	2	10.00%	
41 (lower right central)	Absence	1	5.00%	13	65.00%	<0.001 *
	Presence	2	10.00%	4	20.00%	
33 (lower left canine)	Absence	7	35.00%	11	55.00%	0.125
	Presence	0	0.00%	2	10.00%	
43 (lower right canine)	Absence	2	10.00%	16	80.00%	0.031 *
	Presence	0	0.00%	2	10.00%	
35 (lower left 2nd premolar)	Absence	4	20.00%	8	40.00%	0.001 *
	Presence	2	10.00%	6	30.00%	
45 (lower right 2nd premolar)	Absence	13	65.00%	6	30.00%	0.031 *
	Presence	0	0.00%	1	5.00%	

* significant at the 0.05 level. T0: before starting the treatment. T2: after finishing leveling and alignment.

**Table 9:** Comparison of the amount of change in the alveolar crest height during (T0–T2) between the two groups according to the teeth studied.

Tooth	I-Arch Group		Control Group		Differences Between the Two Means	*p*-Value
	Mean	SD	Mean	SD		
11	−0.01	0.82	−0.39	0.67	0.38	0.121 ^a^
21	−0.17	0.68	−0.36	0.78	0.19	0.427 ^a^
13	−0.07	1.12	−0.47	0.79	0.4	0.203 ^a^
23	0.02	1.3	−1	1.81	1.02	0.042 ^b,^*
15	−0.95	1.93	−0.54	1.53	−0.41	0.745 ^b^
25	−0.47	1.47	−1.07	2.04	0.6	0.498 ^b^
31	−3.64	2.73	−3.89	3.02	0.25	0.781 ^a^
41	−3.3	3.58	−3.8	3.37	0.5	0.649 ^a^
33	−0.78	3.1	−1.97	4.03	1.19	0.533 ^b^
43	−0.95	2.12	−1.49	2.16	0.54	0.588 ^b^
35	1.24	4.22	−1.12	3.57	2.36	0.064 ^a^
45	1.93	3.12	−2.76	10.09	4.69	0.020 ^b,^*

^a^: Independent *t*-test, ^b^: Mann–Whitney U test. * significant at the 0.05 level. Δ bone Alveolar: the amount of buccal alveolar crest change during treatment (T0–T2).

**Table 10:** Comparison of the amount of change in the alveolar crest height during (T0–T2) between the two groups according to all studied teeth.

	I-Arch Group		Control Group		Differences Between the Two Means	*p*-Value
	Mean	SD	Mean	SD		
Δ bone Alveolar	−0.65	1.06	−1.63	1.29	1.21	0.009 ^b,^*

^b^: Mann–Whitney U test. * significant at the 0.05 level. Δ bone Alveolar: the amount of buccal alveolar crest changes during treatment (T0–T2).

**Table 11:** Comparison of the number and percentage of dehiscence and fenestrations after leveling and alignment between the two groups according to the teeth studied.

Tooth	Dehiscence and Fenestrations (T2)	I-Arch Group		Control Group		*p*-Value
		The Number	Percentage	The Number	Percentage	
11 (upper right central)	Absence	19	95.00%	16	80.00%	0.151
	Presence	1	5.00%	4	20.00%	
21 (upper left central)	Absence	17	85.00%	12	60.00%	0.008 *
	Presence	3	15.00%	8	40.00%	
13 (upper right canine)	Absence	16	80.00%	12	60.00%	0.197
	Presence	4	20.00%	8	40.00%	
23 (upper left canine)	Absence	19	95.00%	12	60.00%	0.018 *
	Presence	1	5.00%	8	40.00%	
15 (upper right 2nd premolar)	Absence	14	70.00%	5	25.00%	0.168
	Presence	6	30.00%	15	75.00%	
25 (upper left 2nd premolar)	Absence	16	80.00%	15	75.00%	0.705
	Presence	4	20.00%	5	25.00%	
31 (lower left central)	Absence	3	15.00%	1	5.00%	0.292
	Presence	17	85.00%	19	95.00%	
41 (lower right central)	Absence	2	10.00%	3	15.00%	0.147
	Presence	18	90.00%	17	85.00%	
33 (lower left canine)	Absence	11	55.00%	7	35.00%	0.256
	Presence	9	45.00%	13	65.00%	
43 (lower right canine)	Absence	0	0.00%	2	10.00%	0.028 *
	Presence	20	100.00%	18	90.00%	
35 (lower left 2nd premolar)	Absence	9	45.00%	6	30.00%	0.204
	Presence	11	55.00%	14	70.00%	
45 (lower right 2nd premolar)	Absence	13	65.00%	13	65.00%	1
	Presence	7	35.00%	7	35.00%	

* significant at the 0.05 level. T1: after finishing leveling and alignment.

**Table 12:** Comparison of the numbers of dehiscence and fenestrations between the two groups according to all studied teeth.

	I-Arch Group		Control Group		Differences Between the Two Means	*p*-Value
	Mean	SD	Mean	SD		
Dehiscence and fenestrations (T0)	5.05	3.55	5	3.54	0.05	0.965 ^a^
Dehiscence and fenestrations (T2)	10.05	2.8	12.65	3.1	−2.6	0.008 ^a,^*
Δ Dehiscence and fenestrations	5	2	7.65	3.07	−2.65	0.001 ^b,^*

^a^: Independent *t*-test, ^b^: Mann–Whitney U test. * significant at the 0.05 level. Dehiscence and fenestrations: the number of dehiscence and fenestrations. Δ Dehiscence and fenestrations: the changed amount of dehiscence and fenestrations during treatment (T0–T2). T0: before starting the treatment. T2: after finishing leveling and alignment.

## Data Availability

The original contributions presented in this study are included in the article. Further inquiries can be directed to the corresponding author.
